# Supplementary material for: Rationalizing the design of a broad coverage Shigella vaccine based on evaluation of immunological cross-reactivity among S. flexneri serotypes
Source: PLoS Negl Trop Dis. 2021 Oct 13;15(10):e0009826. doi: 10.1371/journal.pntd.0009826 (PMC8589205; doi:10.1371/journal.pntd.0009826)
Supplement: S1 Table — (DOCX) [file pntd.0009826.s001.docx]

**S1 Table. Characterization of *S. flexneri* strains used in the study by slide agglutination and FACS typing.**

FACS typing and agglutination of *S. flexneri* strains, received from Public Health England, used Denka Seiken monovalent rabbit typing and grouping sera to confirm identity of the serotypes. Mean Fluorescence Intensities (MFI) from 10 to 100 were considered indicative of absence of signal (no binding of respective antisera to the surface of *S. flexneri* serotypes) and correlated with negative agglutination (-). MFI from 101 to 300 was indicative of a weak signal and correlated with weak agglutination (+/-); MFI from 301 to 20000 was indicative of binding and correlated to medium or strong agglutination (+ or ++ or +++) relative to the magnitude of fluorescence intensity signal.

Type or grouping sera are supplied, adsorbed to remove cross-reacting agglutinins, resulting in serotype or group factor specific reagents. There are probably a multitude of epitopes not covered by the typing, grouping scheme currently in use.

| ***S. flexneri* serotype** | **Type specific serum**  Mean Fluorescent Intensity  Agglutination strength | | | | | | **Grouping serum**  Mean Fluorescent Intensity  Agglutination strength | | | |
| --- | --- | --- | --- | --- | --- | --- | --- | --- | --- | --- |
|  | **I** | **II** | **III** | **IV** | **V** | **VI** | **3(4)** | **6** | **7(8)** |  |
| **1a** | 7500  +++ | 30  - | 50  - | 60  - | 60  - | 40  - | 7450  +++ | 30  - | 40  - |  |
| **1b** | 8100  +++ | 50  - | 60  - | 60  - | 50  - | 60  - | 7200  ++ | 8950  ++ | 30  - |  |
| **1c** | 7900  +++ | 60  - | 150  - | 50  - | 60  - | 40  - | 1550  + | 40  - | 30  - |  |
| **2a** | 50  - | 9200  +++ | 90  - | 60  - | 60  - | 40  - | 5200  +++ | 30  - | 25  - |  |
| **2b** | 70  - | 8700  +++ | 60  - | 60  - | 30  - | 40  - | 30  - | 25  - | 1530  ++ |  |
| **3a** | 30  - | 40  - | 8500  +++ | 60  - | 30  - | 60  - | 1200  +/- | 6250  ++ | 3540  ++ |  |
| **3b** | 50  - | 90  - | 7200  +++ | 50  - | 40  - | 80  - | 30  - | 4500  ++ | 45  - |  |
| **4a** | 80  - | 60  - | 30  - | 7500  +++ | 30  - | 40  - | 2350  +++ | 25  - | 30  - |  |
| **4b** | 90  - | 80  - | 60  - | 7600  +++ | 30  - | 60  - | 20  - | 1250  ++ | 30  - |  |
| **5a** | 30  - | 60  - | 40  - | 80  - | 5800  +++ | 80  - | 6500  +++ | 40  - | 30  - |  |
| **5b** | 250  - | 120  - | 170  - | 30  - | 6600  +++ | 60  - | 40  - | 30  - | 620  +/- |  |
| **6** | 150  - | 190  - | 120  - | 90  - | 60  - | 2400  ++ | 250  +/- | 30  - | 30  - |  |
| **X** | 80  - | 90  - | 110  - | 60  - | 90  - | 60  - | 20  - | 30  - | 450  +/- |  |
| **Y** | 150  - | 180  - | 90  - | 90  - | 90  - | 80  - | 540  +/- | 20  - | 20  - |  |
