# Supplementary material for: Rationalizing the design of a broad coverage Shigella vaccine based on evaluation of immunological cross-reactivity among S. flexneri serotypes
Source: PLoS Negl Trop Dis. 2021 Oct 13;15(10):e0009826. doi: 10.1371/journal.pntd.0009826 (PMC8589205; doi:10.1371/journal.pntd.0009826)
Supplement: S2 Table — (DOCX) [file pntd.0009826.s002.docx]

**S2 Table. Experimentally derived matrix of surface staining of *S. flexneri* serotypes reported as Mean Fluorescence Intensities**

Matrix showing the mean fluorescence intensities of surface staining of *S. flexneri* and *S. sonnei* target bacteria stained with pooled sera raised against GMMA in mice. Binding to homologous serotypes is shown in **bold**. The threshold criterion for binding was MFI ≥ 130 (Log MFI 2.1), 10-fold over the background in our assay. Relevant heterologous cross-reaction (of sera raised against *S. flexneri* 3a GMMA on *S. flexneri* 2a bacteria) used as threshold criterion is highlighted in red. The results of one of two comparable technical replicates is shown

| **Immunizing GMMA** | **1a** | **12500** | **7250** | 185 | 10 | 40 | 20 | 11974 | 5062 | 550 | 50 | 2050 | 10 | 683 |
| --- | --- | --- | --- | --- | --- | --- | --- | --- | --- | --- | --- | --- | --- | --- |
|  | **1b** | **6840** | **8240** | 840 | 5520 | 20 | 10 | 3196 | 1956 | 2500 | 5300 | 1560 | 10 | 403 |
|  | **1c** | **9850** | **6500** | 850 | 1250 | 10 | 10 | 6310 | 3239 | 1300 | 1000 | 2530 | 10 | 278 |
|  | **2a** | 100 | 20 | **15530** | **12750** | 30 | 10 | 425 | 137 | 10 | 10 | 150 | 10 | 306 |
|  | **2b** | 30 | 10 | **45430** | **98520** | 10 | 30 | 570 | 197 | 150 | 1250 | 450 | 10 | 280 |
|  | **3a** | 60 | 4650 | 130 | 10 | **76520** | **25540** | 1372 | 394 | 150 | 50 | 350 | 10 | 417 |
|  | **3b** | 450 | 15540 | 100 | 10 | **45840** | **58390** | 812 | 97 | 220 | 590 | 250 | 10 | 296 |
|  | **4a** | 1050 | 450 | 450 | 10 | 10 | 10 | **17997** | 3923 | 480 | 85 | 2450 | 10 | 211 |
|  | **4b** | 50 | 20 | 10 | 60 | 150 | 300 | **133** | 77 | 10 | 75 | 90 | 10 | 19 |
|  | **5a** | 3650 | 40 | 150 | 30 | 10 | 10 | 9440 | **9958** | 560 | 250 | 950 | 10 | 199 |
|  | **5b** | 240 | 20 | 20 | 7820 | 28530 | 10 | 8667 | **4508** | 250 | 1220 | 550 | 10 | 260 |
|  | **6** | 180 | 70 | 70 | 20 | 40 | 40 | 1777 | 1125 | **20700** | 40 | 70 | 10 | 226 |
|  | **X** | 250 | 30 | 10 | 10 | 20 | 10 | 4211 | 4546 | 180 | **540** | 350 | 10 | 190 |
|  | **Y** | 1200 | 20 | 40 | 10 | 10 | 50 | 8614 | 8852 | 220 | 10 | **3250** | 10 | 786 |
|  | **Ss** |  |  |  |  |  |  |  |  |  |  |  | **12500** | **40710** |
|  | **2a OAg-** | 60 | 45 | 30 | 65 | 70 | 85 | 60 | 45 | 80 | 65 | 65 | 45 | 6300 |
|  | | **1a** | **1b** | **2a** | **2b** | **3a** | **3b** | **4a** | **5b** | **6** | **X** | **Y** | **Ss** | **Ss OAg-** |
|  |  | **Target bacterial strain** | | | | | | | | | | | | |
