# Supplementary material for: Rationalizing the design of a broad coverage Shigella vaccine based on evaluation of immunological cross-reactivity among S. flexneri serotypes
Source: PLoS Negl Trop Dis. 2021 Oct 13;15(10):e0009826. doi: 10.1371/journal.pntd.0009826 (PMC8589205; doi:10.1371/journal.pntd.0009826)
Supplement: S3 Table — (DOCX) [file pntd.0009826.s003.docx]

**S3 Table. Experimentally derived Serum Bactericidal Activity (SBA) Titers reported at IC50.**

Matrix showing SBA titers on *S. flexneri* and *S. sonnei* target bacteria with the pooled sera raised in mice against GMMA. Titers on homologous serotypes are shown in **bold**. % BRC: Percent of baby rabbit complement added in the SBA assay. The threshold criteria for killing was defined as IC_50_ ≥ 500 (Log IC_50_ 2.7), 5-fold higher the minimum measurable titer of 100 in our assay. An IC_50_ = 500 was chosen as the threshold of cross-functionality for relevant SBA responses.

| **Immunizing GMMA** | **1a** | **61730** | **62779** | 2270 | 10 | 10 | 10 | 173154 | 39576 | 5411 | 618 | 10472 | 10 |
| --- | --- | --- | --- | --- | --- | --- | --- | --- | --- | --- | --- | --- | --- |
|  | **1b** | **5844** | **4984** | 7560 | 51582 | 10 | 10 | 1003208 | 6914 | 8814 | 42844 | 13094 | 10 |
|  | **1c** | **5554** | **1176** | 8271 | 51272 | 10 | 10 | 2020060 | 9653 | 8323 | 10958 | 20995 | 10 |
|  | **2a** | 1009 | 10 | **62313** | **104306** | 10 | 10 | 159426 | 1650 | 10 | 10 | 948 | 10 |
|  | **2b** | 10 | 10 | **334660** | **511451** | 10 | 10 | 59787 | 2900 | 1001 | 8100 | 2696 | 10 |
|  | **3a** | 10 | 53119 | 1033 | 10 | **106335** | **125249** | 627983 | 20820 | 1194 | 10 | 2715 | 10 |
|  | **3b** | 2739 | 40955 | 1014 | 10 | **276484** | **243798** | 1676371 | 3903 | 1945 | 4085 | 2547 | 10 |
|  | **4a** | 6658 | 10 | 3399 | 10 | 10 | 10 | **152145** | 7475 | 3302 | 732 | 16870 | 10 |
|  | **4b** | 555 | 10 | 10 | 697 | 1035 | 185 | **112804** | 1513 | 10 | 755 | 820 | 10 |
|  | **5a** | 13966 | 10 | 1141 | 10 | 10 | 10 | 750632 | **457295** | 7401 | 2276 | 8303 | 10 |
|  | **5b** | 2647 | 10 | 942 | 81134 | 66789 | 10 | 312004 | **75871** | 2457 | 10 | 5412 | 10 |
|  | **6** | 372900 | 4243 | 178 | 10 | 10 | 10 | 116782 | 10461 | **131323** | 21149 | 3257 | 10 |
|  | **X** | 1835 | 10 | 10 | 10 | 10 | 10 | 48647 | 4705 | 728 | **4294** | 2122 | 10 |
|  | **Y** | 6077 | 10 | 10 | 10 | 10 | 10 | 96609 | 30544 | 1262 | 10 | **4989** | 10 |
|  | **Ss** |  |  |  |  |  |  |  |  |  |  |  | **2850** |
|  | **Sonflex**  **1-2-3** | 168206 | 1790664 | 462706 | 30648 | 373423 | 119785 | 27352 | 7037 | 1164 | 1290 | 9231 | **7943** |
|  |  | **1a** | **1b** | **2a** | **2b** | **3a** | **3b** | **4a** | **5b** | **6** | **X** | **Y** | **Ss** |
|  | % BRC | 10 | 15 | 15 | 7.5 | 15 | 15 | 7.0 | 7.0 | 25 | 7.5 | 7.5 | 20 |
|  |  | **Target bacterial strain** | | | | | | | | | | | |
