# Supplementary material for: Rationalizing the design of a broad coverage Shigella vaccine based on evaluation of immunological cross-reactivity among S. flexneri serotypes
Source: PLoS Negl Trop Dis. 2021 Oct 13;15(10):e0009826. doi: 10.1371/journal.pntd.0009826 (PMC8589205; doi:10.1371/journal.pntd.0009826)
Supplement: S4 Table — (DOCX) [file pntd.0009826.s004.docx]

**S4 Table. OAg to Protein ratios and OAg population of *S. flexneri* GMMA from the different serotypes**

OAg in the GMMA was quantified by HPAEC-PAD measuring rhamnose content. GMMA total protein was determined using a micro-BCA assay kit and bovine sera albumin for the standard curve. The profile and number of OAg repeating units (RU) of the extracted LPS of the immunizing GMMAs compared to the wild-type *S. flexneri* bacteria of the same serotype were similar as judged by SDS-PAGE.

| ***S. flexneri* GMMA serotype** | **OAg/GMMA-Protein ratio (w/w) *** | **OAg molecular weight OAg**  **(MMW = 14 - 20 RU)**** |
| --- | --- | --- |
| **1a** | 0.39 | MMW |
| **1b** | 0.58 | MMW |
| **1c** | 0.53 | MMW |
| **2a** | 0.42 | MMW |
| **2b** | 0.80 | MMW |
| **3a** | 0.48 | MMW |
| **3b** | 0.39 | MMW |
| **4a** | 0.39 | MMW |
| **4b** | 0.48 | MMW |
| **5a** | 0.38 | MMW |
| **5b** | 0.58 | MMW |
| **6** | 0.39 | MMW |
| **X** | 0.12 | MMW |
| **Y** | 0.46 | MMW |

* The OAg concentration quantified in purified GMMA ranged from 0.619 mg/mL for *S. flexneri* 2b to 0.100 mg/mL for *S. flexneri* X. The free protein content of the different serotype GMMAs was <10%.

**The number of OAg repeating units was estimated from LPS pattern on silver stained SDS-PAGE.
