# Supplementary material for: Rationalizing the design of a broad coverage Shigella vaccine based on evaluation of immunological cross-reactivity among S. flexneri serotypes
Source: PLoS Negl Trop Dis. 2021 Oct 13;15(10):e0009826. doi: 10.1371/journal.pntd.0009826 (PMC8589205; doi:10.1371/journal.pntd.0009826)
Supplement: S1 Fig — (DOCX) [file pntd.0009826.s005.docx]

**S1 Fig.** Antisera generated against OAg negative GMMA were unable to bind to OAg positive bacteria


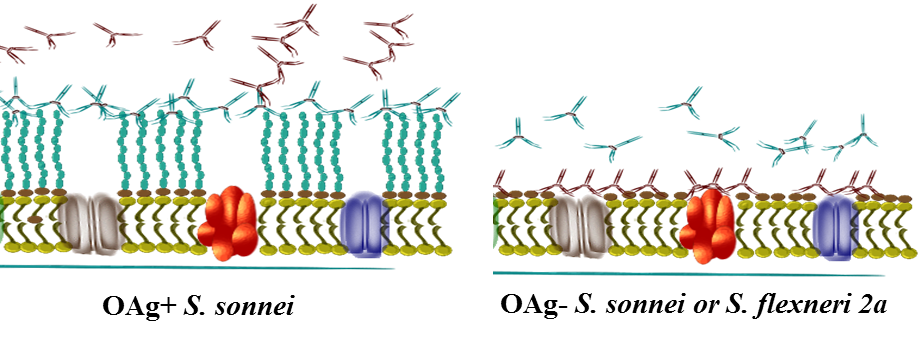


Based on the SBA and FACS binding data, antibody (maroon) directed against common *S. sonnei* and *S. flexneri* proteins and LPS core structures cannot access the surface of the outer membrane in bacteria with intact O-antigen (left panel) because of steric hindrance of O-antigen (teal) and/or antibodies to O-antigen but, these antibodies can bind to both *S. sonnei* and *S. flexneri* 2a that do not express O-antigen (right panel).
