# Supplementary material for: Rationalizing the design of a broad coverage Shigella vaccine based on evaluation of immunological cross-reactivity among S. flexneri serotypes
Source: PLoS Negl Trop Dis. 2021 Oct 13;15(10):e0009826. doi: 10.1371/journal.pntd.0009826 (PMC8589205; doi:10.1371/journal.pntd.0009826)
Supplement: Attachment — Submitted filename: Citiulo_ShigCrossreact_20210906_Letter.docx [file pntd.0009826.s006.docx]

06/09/2021

Dear Drs Christian E. Demeure and Javier Pizarro-Cerda,

Thank you for sharing the Reviewers’ comments on our manuscript. We appreciate the Reviewer’s time and constructive feedback and are happy that the editorial Board and the Reviewers think that our manuscript is of interest.

We have addressed the requested changes to the text and presentation of data to satisfy the reviewers’ needs, resulting in a significantly improved document that we are resubmitting for your reconsideration. As requested, we are providing two version of the manuscript: a clean version and one with track changes. Please find below our point-to-point response to reviewers’ comments and requests. The Reviewers’ comments and requests are in black, followed by the GVGH response in blue.

We believe the findings are of importance to the *Shigella* vaccine community as highlighted by both the reviewers and merit publication in PLOS Neglected Tropical Diseases.

Please note, the email address provided for Laura Martin will only be operational until 30 September 2021; after that date the corresponding author will be Francesco Citiulo.

We look forward to receiving your decision on this improved paper.

Sincerely,

| **Laura B. Martin**  **Senior Advisor & Typhi/Paratyphi A vaccine lead**  **GSK Vaccines Institute for Global Health S.r.l.,**  Via Fiorentina, 1, 53100 Siena, Italy  **Email:**  laura.b.martin@gsk.com | **Francesco Citiulo**  **Scientist II**  **GSK Vaccines Institute for Global Health S.r.l.,**  Via Fiorentina, 1, 53100 Siena, Italy  **Email:**   francesco.x.citiulo@gsk.com |
| --- | --- |

**Detailed response to the review comments and description of changes (PNTD-D-21-00607)**

**Methods**

Reviewer #1: (No Response)

Reviewer #2:

This is a preclinical study to assess cross-reactive immune responses, including functional antibodies, in mice (and rabbits) to a 14 different Shigella GMMA vaccine constructs.

The heat map methodology is an important element of the paper but is not easy to follow.

line 201 does this whole explanation relate to the predicted IC50 shown in fig 2C? if so please clarify, and that the section on line 188 relates to the measured SBA in 2B.

To help clarify differences among the three original heat maps in Figure 2, the authors have split this figure into two. Figure 2 now contains the experimentally derived data originally presented as Fig 2*A* (FACS) and Fig 2*B* (SBA; starting at original line 188; now lines 196-210).

A new Figure 3, now contains the theoretically derived antibody cross-functionality based on defined typing specificities. Figure 3*A* provides the basis of the theoretical model as originally described starting at line 201 (now lines 213-215) and Fig. 3*B* presents the modelled heat map (as explained now starting from line 215).

To clarify the distinction between experimental and theoretical cross-reactivity, we have also modified the section headers as follows:

- Original line 170 (now line 177): Cross-reactivity of anti-GMMA sera measured by FACS
- Original line 188 (now line 196): Cross-functionality of anti-GMMA sera measured by high through-put Luminescence - Serum bactericidal assay
- Original line 201 (now line 212): Theoretical SBA heat map modelled on typing sera specificities

Corresponding updates were made to the Figure legends as well.

line 203: should this be representative heat map (if referring to methods for fig 1 A), not a theoretical heat map? since theoretical heat map is 1B?

We hope that the changes made in response to the previous comment helps to differentiate the experimentally derived data sets (Fig. 2) from these theoretical SBA results derived from classical typing antibody specificities (Fig. 3). Additional wording has been added to this section (lines 213-216) to better define the assumptions (visualized in new Fig 3*A*) used to define the theoretical SBA heat map (Fig 3*B*).

line 206: please clarify how the cut of of 3.9 for heterologous SBA was derived? is this the 'maximum' observed?

We have further clarified that the threshold of 3.9 as referring to theoretical SBA heat map (Fig 3*B*); the threshold was derived from the average Log(IC50) of heterologous serotypes sharing a single common typing specificity (current line 219). These specificities are now clearly shown as part of the assumptions presented in new Fig 3*A*.

Struggled to follow the section on modelled heat map - like 201 onwards.

The authors apologize for the lack of clarity in generating the modelled theoretical heat map. Starting from new line 213-218, additional explanation has been provided as well as part of the new added Fig 3*A* that contains the theoretical matrix of cross-reactivity mediated by serotype and group specificities shared among the immunizing GMMA and the target bacterial strain. Implementing Fig 3*B* (previously Fig 2*C*) where the observed Log IC50 of sera tested on the homologous and heterologous serotypes were used to assign an hypothetical strength of cross-functionality in building the heat map.

The abstracts mentions that the study includes rabbits, but no mention is made here.

Based on suggestion of both reviewers 2 and 3, supplemental data Fig S1 in the previous submission has now been included as new Figure 4. To support this addition, lines 170-175 have been included in the methods to describe the rabbit immunogenicity study. Reference to Figure 4 has been added throughout the text.

Reviewer #3:

1. Line 201- the description of the construction of the modeled SBA heatmap is unclear.

Based on suggestion of both reviewers 2 and 3 additional explanation has been provided (new line 213-218), as well as part of the new added Fig 3. Please see the response and associated modifications provided to Reviewer #2 (above).

2. Please comment on the chain length for the OAg produced by the GMMA vaccines.

Additional characterization, including number of OAg repeating units, estimated from LPS pattern on silver stained SDS-PAGE, of the GMMA used in this study has been included in supplemental data Table S4.

3. It appears that different amounts of complement have been used for the measurements of bactericidal activity against various Shigella serotypes (Table S3). This can be a confounder in the interpretation of the results. How were the assays harmonized for valid comparison across strains?

Different bacterial lines, strains and species inherently possess different sensitivities to complement. This was taken into consideration in the L-SBA development, curve fitting and data analysis, as described in ref [25]. For each bacterial strain, we initially determined the optimal lot and percentage of baby rabbit complement (BRC) to be used. From the titration of BRC against each bacterial line, the highest % active BRC that lacked intrinsic bactericidal activity was selected for testing of antisera. Additionally, each assay contained the same negative control (active BRC and heat inactivated BRC without any antisera). Raw luminescence values (LUM) from each bactericidal reaction in the presence of test serum (LUMtest180) were normalized to the maximum bacterial growth obtained after 3 h (180 min, T180) incubation (LUMmax180), by calculating LUMtest180/LUMmax180. Normalized data were then analyzed by a four-parameter logistic (4-PL) curve fitting model and the reciprocal serum dilution that resulted in a 50% reduction of luminescence normalized for the maximum luminescence signal (eg., the highest serum dilution, equal to the signal given by the complement controls) was reported as the titer of the serum sample. The normalization was convenient to plot multiple data sets on the same scale and had no impact in determining the bactericidal titer. Variation in the maximum growth of different bacteria strains did influence the baseline of the bactericidal curves, which varied between 0.1 (less bacterial growth) and 0.01 (higher bacterial growth).

4. Line 191. Explain why the heat inactivation was done at 54oC for 1h as opposed to the usual 56oC for 30 min.

The authors apologize for the methodologic inaccuracies. Time and temperature in line 191 were updated to indicate “30 min for 56°C”, in line with the methodology cited, originally ref [24](now ref. [25]) Necchi et al 2017 (PMID: 28192483) showing utility on *S. sonnei* and *S. flexneri* serotypes.

--------------------

**Results**

Reviewer #1: (No Response)

Reviewer #2: line 235: threshold of cross reactivity in FACS analysis was assigned based on a historical preclinical challenge study that assessed 2 serotypes: flex 3a and 2a. this is presented as a threshold that is 'predictive of field cross coverage'. suggest to include reference to limitations of this extrapolation, including that it is based on only one heterologous assessment. essentially this is one benchmark.

The reviewer is correct that the FACS threshold is based on limited data. The text for this section (new lines 251-258) has been revised to provide greater clarification for the selection of threshold (MFI ≥ 130 or Log MFI 2.1). By analogy with the Noriega et al. protection data (reference 13), this threshold value was derived from our experimental FACS data of *S. flexneri* 3a antisera staining of *S. flexneri* 2a bacteria and possible limitations as well are highlighted.

line 306: 'The breadth of the specificity was a little greater as judged by SBA than FACS' - this seems odd. Could the threshold for  coding the heat maps be lacking in sensitivity?

As indicated in original lines 303-304 (new lines 321-322), there is good concordance between the two assays with few inconsistencies. Methodologic differences may have contributed to the variation observed between FACS and SBA results, including:

- Fixation of the bacteria as part of the FACs analysis may have slightly altered an epitope thus reducing binding of antibodies to the bacteria.
- The output of the L‑SBA is more quantitative as it relies on a larger dilution curve of sera compared a single optimized dilution assessed by FACS.
- The presence of a low abundant, highly potent bactericidal antibody, that due to it concentration gives low / near background signal in FACS.

line 316: selection of 1b and 3a based on cross reactivity data:  It is not clear why, on the basis of figures 2A and 2B, these sequences were selected. for example, 1c has very similar values  to 1b? 3b actually has higher values than 3a in many instances for FACS, but not so for SBA. please expand basis/rationale for serotype selection line

Selection of the *S. flexneri* GMMA serotypes to include in the multicomponent vaccine was not based only on our cross-binding and cross-functional activity datasets but also on the currently accepted understanding of the serotype epidemiology contributing to shigellosis. A phrase in this regard has been added (new line 344-346) and further text has been added to the discussion (new lines 457-459) for the inclusion of *S. flexneri* 1b over *S. flexneri* 6.

319: would be useful to mention why sonnei was included based on the results - obvious, but a statement on this would be helpful nonetheless.

An additional sentence was added (new lines 345-346), indicating that the *S. sonnei* immunogen was included in the multicomponent *Shigella* vaccine composition due to its contribution to the global disease burden. In the context of this study, *S. sonnei* GMMA antisera or test bacteria for FACS or SBA was included as a control for other potentially cross-reactive components of the GMMA (further emphasized by addition of phrase to new line 187).

line 323 mention of rabbit results being supportive but there is not mention of the rabbit study throughout (apart from abstract). suggest to remove.

As indicated in the “Methods section” response, based on suggestion of both reviewers 2 and 3, the rabbit data originally included as supplemental data Fig S1 has now been included as new Figure 4.

Reviewer #3:

1. A major concern is that the GMMAs contain molecules other than LPS (e.g. outer membrane proteins) and therefore cross reactivity may not be due to O-antigen alone. Sera directed against GMMA from O-negative strain (Line 279) was used to conclude that the antibodies examined are directed against the LPS only (Figure 2S). However, there is no information to indicate the protein composition of the different GMMAs.

The protein composition of *S. sonnei* and *S. flexneri* GMMA has been previously published, Maggiore et al (reference [16]) and is cited in the discussion (original line 344, now line 365-366).

Since the original submission of this manuscript, GVGH has published results of the recognition of *Shigella* bacterial proteins by GMMA antisera (mouse and human) and the impact on SBA responses. As described by Mancini et al. 2021 (ref. [26]) and Frenck RW et al. 2021 (ref. [20]), anti-OAg antibodies elicited by OAg positive GMMA were the main drivers of bactericidal activity against OAg-positive bacteria, while antibodies not targeting the OAg are functional only against OAg-negative bacteria. Sentences and associated references have been added (to the results in new lines 299-302 and to the discussion in new lines 382-391).

According to Table S4, the GMMAs have quite different O/protein ratios. A more convincing demonstration of specificity (for example, complete O antigen inhibition of reactivity) would be needed.

Based on the recently published data described above (Mancini et al. 2021 (ref. [26]) and Frenck RW et al. 2021 (ref. [20])), the authors do not believe further demonstration of specificity (eg by adsorption of sera) is necessary.

2. The claim that a vaccine containing such serotypes can be broadly protective needs to be confirmed by protective efficacy in vivo.

Both the intent to evaluate cross-reactivity using human sera coming from ongoing or planned clinical trials of the 4 component *Shigella* vaccine and the inherent limitations of preclinical animal models for in vivo protection against *Shigella* have been added to the discussion (new lines 450-456)

3. The supplementary material has important information that could be better appreciated within the main manuscript. A re-evaluation of all figures is in order; see comment below.

Thank you for this suggestion, as indicated in previous responses data previously included as supplementary material has been moved to the main text (new Fig. 4).

4. In Lines 317-319, the authors indicate that humans recognize some Shigella serospecificities differently to mice. In Figure S1, the authors show that sera from rabbits immunized with S. flexneri 2a GMMA elicited SBA against S. flexneri 6, which is similar to what has been observed in humans. Since the rabbit data may more closely resemble human data, Figure S1 seems more relevant than Figure 2.

Again, thank you for this suggestion, as indicated in previous responses the prior Fig. S1 has now been included in the main text as Fig. 4.

5. It is not clear the message the authors are trying to convey with the predictions in Figure 2C. Please explain the value of this prediction.

By adding additional interpretation of results (new lines 333-338), we have highlighted observed heterologous cross-reactions that would not have been predicted from the dogma of defined group specificities.

Overall, as stated in the abstract and discussion, we revisited the current view of cross-reactivity among *S. flexneri* serotypes and identified broad-specificity immunogens. Thus, supporting the feasibility of a broadly protective vaccine with a limited number of appropriately selected components.

6. Line 235- The authors cite two studies (not mouse or rabbit) when defining the threshold criterion. Please provide more detail on how a threshold criterion was determined, especially without challenge data.

We have explained better the determination of the threshold and the limitation regarding its use (actual lines 250-255)

7. Figure S1 (A). Check for consistency in format.  Please comment on the comparison of SBA titers if different amount of complement were used.

Thank you for spotting the inconsistencies in formatting, which we have addressed. For explanation on use of different amounts of complement and normalization across bacterial strains, please see response under the “Methods section” to Reviewer #3 point 3. We hope the explanation provided above for the standardization of the SBA assay across different strains and inclusion of the appropriate SBA reference (Necchi et al 2017 (ref. [25]) replacing Necchi et al 2018) are sufficient.

--------------------

**Conclusions**

Reviewer #1: (No Response)

Reviewer #2: The paper concludes that the combination of 4 serotypes identified will provide broad cross reactivity across circulating Shigella, but doesn't contextualise this within the other Shigella serotypes beyond those assessed in this paper.  Could some comment be made as to the clinical/epidemiological relevance of the other Shigella serotypes and the expected impact from this combination?

We have added an additional sentence to the discussion to refocus attention on current endemic *Shigella* serotypes of importance (new lines 440-442 and 458-463). Neither *S. dysenteriae* nor *S. boydii* have been mentioned as they are minor proportion (≤5%) of overall shigellosis globally. Previously, these species caused outbreaks which currently have inexplicably disappeared (Kerneis 2009 and Bardhan 2010).

Mention whether preclinical challenge studies were contemplated to confirm in vitro analysis - if not, why not?

Please refer to prior response on utility of preclinical animal models and additional text added to the discussion (new lines 452-456)

other Shigella combo approaches have selected serotype 6, rather than 1b.  could the authors comment on the relevance/implications of this, based on their analysis?

Please see the additional text added to the discussion (new lines 460-463)

would be helpful for the authors to mention the limitations/assumptions underpinning this analysis and their conclusions line 408: would be helpful to outline the cross over studies that should be done with human studies where sera are available

The planned analysis for antibody cross-reactivity using human sera from ongoing studies has now been included in the discussion as new lines 450-451.

Reviewer #3: 1. While the predicted SBA heatmap (Figure 2C) shows that Noriega et al’s bivalent 2a and 3a and the vaccine proposed by Livio et al (S. flexneri 2a, 3a, 6 and S. sonnei) would not protect, Figure 2B predicts that both would be protective, and even more so, Figure S1. Please address this observation in the discussion.

We did not mean to convey that a combination vaccines proposed by Noriega or Livio would not protect, but rather that perhaps a more broadly protective vaccine could be achievable (with the same number of components) if unrecognized cross-reactions were taken into consideration. In fact, Barry and Levine have recently suggested that *S. flexneri* 1b could be useful addition to a multicomponent Shigella vaccine (2019 Cell Microbiol). This concept has been addressed by adding new text to the discussion (new lines 460-464).

2. While the inclusion of the S. sonnei GMMA is helpful as a proof-of-concept, it is not clear why it was included in the vaccine, as the manuscript is mainly about cross-protection of S. flexneri.

As previously mentioned, the inclusion of *S. sonnei* is based on the current epidemiology of shigellosis, with ±24% of cases resulting from infection with this species. This concept has been enhanced in several places throughout the text.

3. It is unexpected that the bactericidal activity was not the highest for the homologous sera and serotype. Higher titers were obtained in a cross-reactive manner. Again, can this be an artifact of the experimental assay conditions? This issue should be addressed/discussed.

This was observed only with few bacterial strains, most evident on *S. flexneri* 4a; intrinsic complement sensitivity and/or sensitivity of strains to antibody mediated killing might have played a role in this finding. Regardless, the SBA was well above the threshold defined for relevant antibody functionality.

4. In vivo protective data would be important to support the broad coverage conclusions.

Please see our previous responses on why preclinical infection models were not employed and our plans to address cross-reactive antibody responses using human sera (the most appropriate animal model); this concept is provided in lines 452-456.

--------------------

**Editorial and Data Presentation Modifications?**

Reviewer #1: (No Response)

Reviewer #2:   
Line 18: mentions rabbits, but not included in description of study in line9, or the rest of the paper.  suggest to reconcile/remove line 19: add...'evaluated in this study' after flexneri serotypes line 25: suggest to add 'cost' to complexity line 30: basis, not bases line 53: suggest to change this to 'These data highlight the difficulties... if based solely on the basis of epidemiology' - as more relevant to the narrative that proceeds it.  The difficulty of selection based on subtype/serotype specificity could be positioned around line 82.

line 56: change to homologous AND cross reactive serotypes line 57: remove 'whereas'

line 75: remove 'have been'

line 77: Suggest to include This 'selection of sub-types on the basis on group specificity' was the basis for studies....

line 92: suggest change 'the same' to homologous and heterologous line 291: enter 'than' the minimum criterion...

line 347: suggest change instead for conversely

Thank you for these suggestions which have been updated in the text of the paper.

The figures in the results are loaded back to front; 1 is 2 and vice versa

We apologize for the error in uploading the figures. This has been addressed in the current submission.

Reviewer #3:

1. Figures 1 and 2 are inverted. Images of higher resolution should be provided. Also, the figure legends should be self explanatory (i.e. shading explained). The Figures 1 and 2 heatmaps are not easy to interpret. Using graphs in the Supplementary Figure S1 (with actual titers) is recommended for ease of visualization and interpretation.

We apologize for the error in uploading the figures.

Additional guidance and explanation of the heat maps have been added to figure legends of this resubmission. The use of heatmaps was deemed necessary for streamlining the presentation and interpretation of a very large dataset. If the reviewer thinks that the numerous graphs (i.e. bar charts with IC50 for each antisera against tested bacteria) are necessary, the full dataset could be added as part of the supplementary information.

2. Table S3 check for inconsistencies in font (X and Y vs. x and y).

Thank you for spotting the inconsistencies in formatting, which we have addressed.

--------------------

**Summary and General Comments**

Reviewer #1: (No Response)

Reviewer #2: This is an important manuscript that seeks to provide the rationale for the selection of serotypes and subtypes for inclusion in a broadly cross reactive Shigella vaccine.  The paper reads well but could benefit from improved clarify in some areas, as identified above. THANK YOU!

The abstract mentions that the study extended to assess the immunogenicity in rabbits but no mention is made in the rest of the paper, except to support conclusions.  Suggest to expand or remove.

Thank you for the positive feedback and we hope to have improved the readability based on the reviewers comments. As suggested we have moved Fig S1 into the main text where it is now Fig 4.

Reviewer #3: This manuscript describes the capacity of mouse antibodies elicited by vaccination with outer membrane vesicles (exosomes known as GMMA) from Shigella sonnei, to bind S. sonnei and a panel of 11 different S. flexneri subtypes. GMMAs contain all the outer membrane components of the bacteria from which they are derived and may be engineered to express a modified O antigen. Antibody reactivity was determined in vitro via FACS and bactericidal activity. The broader objective of this work is to generate information that would allow to design a vaccine that provides broad coverage against multiple serotypes.

Mouse antisera against S. flex serogroup 1 and 3 (specifically serotype 1b and 3a) recognized most  of S. flexineri serotypes, indicating a vaccine candidate that contains these two serotypes could yield a broad reactivity/coverage. These results are in line with the vaccine proposed by Livio et al., with similar number of serotypes (4) and differing by one serotype, replacing S. flexneri 6 with S. flexneri 1b.

The analysis of Shigella O-antigen cross reactivity is a valid and important question. Please see specific comments under Methods, Results, and Conclusions.

Thank you for the positive feedback. We hope to have improved the communication of the concept that through appropriate selection of the vaccine antigens a higher level of protection can be achieved by exploiting previously unrecognized cross-reactivity and use of broad specificity immunogen (eg, replacing *S. flexneri* 6 with *S. flexneri* 1b).

--------------------
